# Protective Role of a Donepezil-Huprine Hybrid against the β-Amyloid (1-42) Effect on Human Erythrocytes

**DOI:** 10.3390/ijms22179563

**Published:** 2021-09-03

**Authors:** Pablo Zambrano, Mario Suwalsky, Malgorzata Jemiola-Rzeminska, María José Gallardo-Nelson, Kazimierz Strzalka, Diego Muñoz-Torrero

**Affiliations:** 1 Facultad de Ciencias Químicas, Universidad de Concepción, Concepción 4030000, Chile; 2 Facultad de Medicina, Universidad Católica de la Santísima Concepción, Concepción 4030000, Chile; msuwalsky@ucsc.cl; 3 Malopolska Centre of Biotechnology, Jagiellonian University, 30-387 Kraków, Poland; malgorzata.jemiola-rzeminska@uj.edu.pl (M.J.-R.); kazimierz.strzalka@uj.edu.pl (K.S.); 4 Faculty of Biochemistry, Biophysics and Biotechnology, Jagiellonian University, 30-387 Kraków, Poland; 5 School of Medicine, University of Atacama, Copiapó 1530000, Chile; mariajose.gallardo@uda.cl; 6 Laboratory of Medicinal Chemistry (CSIC Associated Unit), Faculty of Pharmacy and Food, Sciences, University of Barcelona (UB), E-08028 Barcelona, Spain; dmunoztorrero@ub.edu; 7 Institute of Biomedicine (IBUB), University of Barcelona (UB), E-08028 Barcelona, Spain

**Keywords:** AVCRI104P4, beta-amyloid, cell membrane, lipid bilayer, human erythrocyte, acetylcholinesterase, multitarget agent

## Abstract

Aβ(1-42) peptide is a neurotoxic agent strongly associated with the etiology of Alzheimer’s disease (AD). Current treatments are still of very low effectiveness, and deaths from AD are increasing worldwide. Huprine-derived molecules have a high affinity towards the enzyme acetylcholinesterase (AChE), act as potent Aβ(1-42) peptide aggregation inhibitors, and improve the behavior of experimental animals. AVCRI104P4 is a multitarget donepezil-huprine hybrid that improves short-term memory in a mouse model of AD and exerts protective effects in transgenic *Caenorhabditis elegans* that express Aβ(1-42) peptide. At present, there is no information about the effects of this compound on human erythrocytes. Thus, we considered it important to study its effects on the cell membrane and erythrocyte models, and to examine its protective effect against the toxic insult induced by Aβ(1-42) peptide in this cell and models. This research was developed using X-ray diffraction and differential scanning calorimetry (DSC) on molecular models of the human erythrocyte membrane constituted by lipid bilayers built of dimyristoylphosphatidylcholine (DMPC) and dimyristoylphosphatidylethanolamine (DMPE). They correspond to phospholipids representative of those present in the external and internal monolayers, respectively, of most plasma and neuronal membranes. The effect of AVCRI104P4 on human erythrocyte morphology was studied by scanning electron microscopy (SEM). The experimental results showed a protective effect of AVCRI104P4 against the toxicity induced by Aβ(1-42) peptide in human erythrocytes and molecular models.

## 1. Introduction

Beta-amyloid peptides (Aβ) have been associated with cell membrane damage, neuroinflammation, neuronal death, synaptic impairment, and memory dysfunction [1,2]. An increasing body of evidence coming from genetic, histopathological, and cell culture studies points to Aβ(1-40) and Aβ(1-42) peptides as the main neurotoxic agents in Alzheimer’s disease (AD) [3,4,5,6]. Aβ fragments are normal products of the enzymatic cleavage (via β- and γ- secretases) of the transmembrane amyloid precursor protein (APP) with broad cell expression [7,8,9,10]. Aβ peptides generally contain 28 amino acid residues from the extracellular portion of APP and 11–15 residues from its transmembrane domain [11,12,13,14]. The most abundant peptide is Aβ(1-40) [15,16]. However, the Aβ(1-42) isoform, which constitutes approximately 10% of the total, is the most abundant peptide in senile plaques [17] and has been associated with a potent toxic effect in its oligomeric form [18,19,20]. Whereas most research efforts over the past two decades have been devoted to the discovery of new drugs that are able to halt or delay AD progression, e.g., disease-modifying drugs, mainly targeting Aβ biology, the currently approved drugs only afford a temporary relief of the symptoms. Among the marketed drugs, inhibition of cerebral acetylcholinesterase (AChE) is the most common mechanism of action [21]. This enzyme has three important sites in its structure: the deep catalytic site, the middle or “gorge” site, and the peripheral site. The peripheral site corresponds to a set of residues that participate as a transitory union of the substrate, and it is abundant in aromatic amino acids that lead to ligands such as acetylcholine, transferring them to the deep catalytic site [22]. On the other hand, there are reports that AChE can accelerate the formation of Aβ peptides and their deposition in the brains of patients with AD [23,24,25,26], which might be related to the activation of the enzyme’s peripheral site [27]. This conclusion was attained by inhibiting the peripheral site of the AChE and evaluating the level of aggregation of the amyloid peptide. Thus, it was determined that the amino acidic AChE residues involved in the acceleration of amyloid peptide aggregation should be close to the peripheral site of the enzyme [23]. Therefore, the use of ligands capable of interacting simultaneously with the active and peripheral sites might have important advantages over the use of any known AChE inhibitor. These dual-binding site ligands would have greater AChE inhibitory potency due to their greater affinity and consequently greater efficacy for the symptomatic treatment of AD, and furthermore they would be expected to additionally exhibit neuroprotective effects arising from the inhibition of the AChE-induced Aβ aggregation [28,29,30].

**Figure 1:**
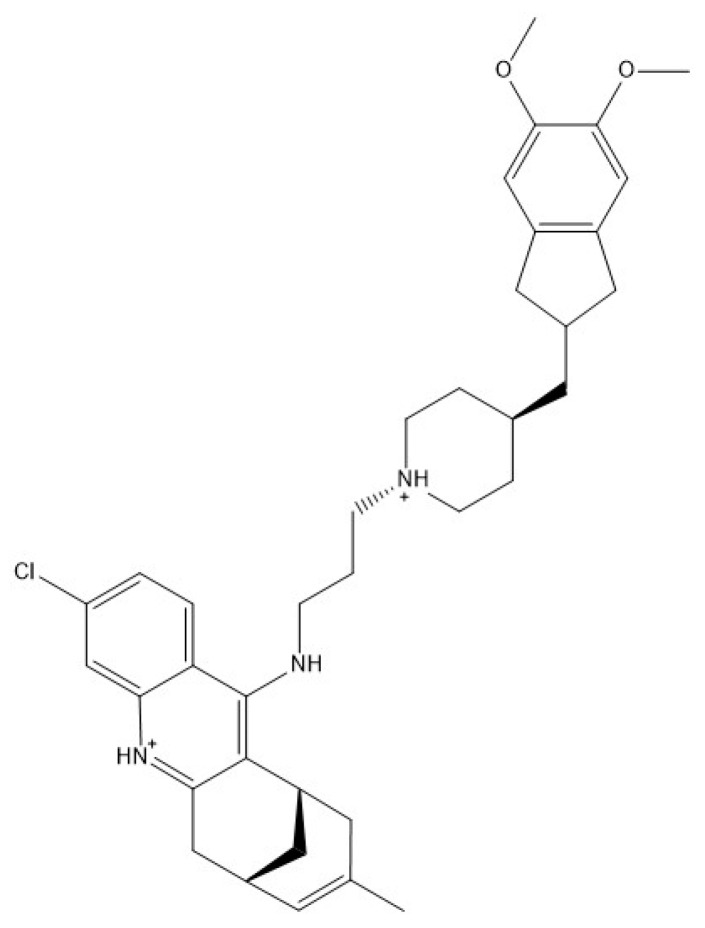
Structural formula of AVCRI104P4.

In order to reduce the development of senile plaques and prevent Aβ peptide aggregation while improving the central cholinergic transmission, several classes of huprine-based dual-binding site AChE inhibitors have recently been developed [31,32,33,34,35]. These compounds behave as multitarget agents as they display potent inhibitory effects against the activity of human AChE (hAChE), human butyrylcholinesterase (hBChE), and also on the in vitro aggregation of Aβ peptides and tau protein. One of these compounds is AVCRI104P4 (Figure 1), a donepezil-huprine hybrid endowed with potent in vitro inhibitory activity of hAChE (IC_50_ = 2.61 nM) and hBChE (IC_50_ = 349 nM), and moderate potency toward BACE-1 (IC_50_ = 11.0 μM) [35]. The ability of AVCRI104P4 to cross the blood–brain barrier (BBB) has been consistently confirmed in a number of in vitro, ex vivo, and in vivo studies. Thus, in vitro determination of its BBB permeability by the widely known parallel artificial membrane permeability assay for BBB (PAMPA-BBB), which uses a lipid extract of porcine brain as a BBB model [36], showed that this compound had a permeability value of 11.4 × 10^−6^ cm s^−1^, above the threshold that indicated good BBB permeation (CNS+, 9.7 × 10^−6^ cm s^−1^) under the employed assay conditions, which was indicative of the fact that this compound should be able to enter the brain [35]. Also, ex vivo determination of brain AChE activity in OF1 mice to which AVCRI104P4 (10 μmol kg^−^^1^) had been previously administered intraperitoneally (i.p.) showed that the enzyme activity was reduced by 59% only 5 min after the i.p. administration of this compound, retaining 46% brain AChE activity inhibition at 20 min after administration [34]. These results indirectly showed that following i.p. administration, AVCRI104P4 rapidly entered the OF1 mouse brain, where it inhibited brain AChE. Finally, in vivo studies in different mouse models treated with either AVCRI104P4 or its racemic form, ACRI104P3, have demonstrated clear beneficial effects on learning, memory, and some neuropsychiatric symptoms, which can only appear if the compound has entered the central nervous system. Thus, AVCRI104P4 (10 to 40 mg/kg/day, 3 months, oral administration) improved short-term memory in APP_SL_ mice [34], whereas chronic treatment of middle-aged (12-month-old) male 129/Sv x C57BL/6 mice with a low dose of AVCRI104P3 (0.43 mg/kg/day, 21 days, i.p.) ameliorated short- and long-term learning and memory and exerted anxiolytic actions [37,38], and elicited neuroprotective effects by increasing cortical and/or hippocampal levels of the anti-apoptotic proteins pAKt1, pGSK3β, and Bcl2, and by reducing microgliosis [39]. The effect of AVCRI104P4 on Aβ aggregation has been studied in vitro and in vivo. By using a thioflavin T fluorescence method [40,41], it was found that AVCRI104P4 inhibited in vitro the aggregation of Aβ(1-40) induced by AChE by 41% at 100 μM and inhibited by 29% the spontaneous aggregation of Aβ(1-42) at a concentration of 10 μM [35]. The rather limited effects of this compound on Aβ aggregation were also found in in vivo studies in *Caenorhabditis elegans* and mouse models of AD. Fluorescence microscopy studies, using the dye X-34, showed that AVCRI104P4 at 100 μM was not able to reduce Aβ(3-42) fibril deposition in CL2006 nematodes, a *C. elegans* strain that forms Aβ(3-42) fibrils and oligomers in the body wall muscle cells [34]. Interestingly, this compound exerted a protective effect on CL4176 nematodes (see below), a *C. elegans* strain that expresses Aβ(1-42), leading to the deposition of oligomers in muscle cells without forming amyloid aggregates. The findings that in none of these strains ACRI104P4 affected the total Aβ levels, as measured by dot blot analyses using the WO2 total Aβ-specific antibody, and that the protective effect of AVCRI104P4 against the Aβ insult was greater in CL4176 than in CL2006 worms seemed to indicate that this compound might be targeting Aβ oligomers and not amyloid plaques or Aβ formation [34]. Consistently with the latter findings, AVCRI104P4 did not alter cortical or hippocampal levels of Aβ peptides (Aβ(1-38), Aβ(1-40), Aβ(1-42)) or amyloid burden in APP_SL_ mice chronically treated with AVCRI104P4, as assessed by 6E10 antibody and thioflavin S staining [34]. As previously mentioned, AVCRI104P4 protected CL4176 and CL2006 *C. elegans* strains from the toxicity (paralysis phenotype) induced by Aβ expression and accumulation [34], with these protective effects being independent of its putative effects on Aβ levels, Aβ fibril deposition, or AChE activity [34], thereby warranting further studies to delineate the mechanism(s) of action of this compound. Herein, we report biophysical studies to shed light on the molecular mechanisms that are behind the protective effect of AVCRI104P4 against the toxicity induced by Aβ, and behind its interaction with cell membranes. To this end, human erythrocytes and molecular models of their membrane consisting of the phospholipids dimyristoylphosphatidylcholine (DMPC) and dimyristoylphosphatidylethanolamine (DMPE) as representative classes of phospholipids located in the external and internal monolayers of the erythrocyte membrane, respectively, were used. X-ray diffraction and differential scanning calorimetry (DSC) were employed to study the interaction of AVCRI104P4 with the multilayers and multilamellar vesicles (MLV) of DMPC and DMPE, respectively. In addition, the morphological effect of AVCRI104P4 on human erythrocytes and its protective effect against the peptide Aβ(1-42) were determined by scanning electron microscopy (SEM).

## 2. Results

### 2.1. Transmission Electron Microscopy (TEM) of Aβ(1-42) Oligomeric Aggregates and Fibers

**Figure 2:**
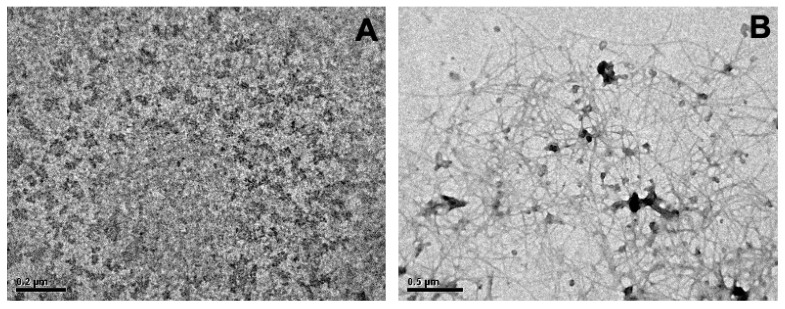
Ultrastructure of Aβ(1-42) oligomers and fiber species. (**A**) Transmission electron micrograph of Aβ(1-42) oligomers (80 μM) obtained after 24 h of aggregation process with a spinning bar at 400 rpm and ∼20 °C (room temperature). (**B**) Micrograph of Aβ(1-42) fibers obtained after the incubation of the oligomeric species for 24 h at 37 °C without agitation.

Transmission electron microscopy experiments were carried out to evaluate the oligomeric and fibrillar ultrastructure of Aβ(1-42). These oligomeric structures were used in subsequent experiments to evaluate the protective effect of AVCRI104P4 against the peptide by X-ray diffraction and scanning electron microscopy (SEM). Figure 2A shows that the Aβ(1-42) soluble oligomers present amorphous structures after 24 h at room temperature. Under these conditions (first 24 h), the soluble Aβ(1-42) monomers are associated in the form of different types of non-fibrillary oligomers, from low molecular weight oligomers such as dimers or trimers to large globular complex combinations [42]. This is consistent with reported observations and morphological characterizations of Aβ(1-42) peptide aggregates [43]. Furthermore, when these oligomers were incubated for an additional time (24 h) at 37 °C, the formation of long and fine fibers was observed (Figure 2B).

### 2.2. X-ray Diffraction of DMPC and DMPE Multibilayers

**Figure 3:**
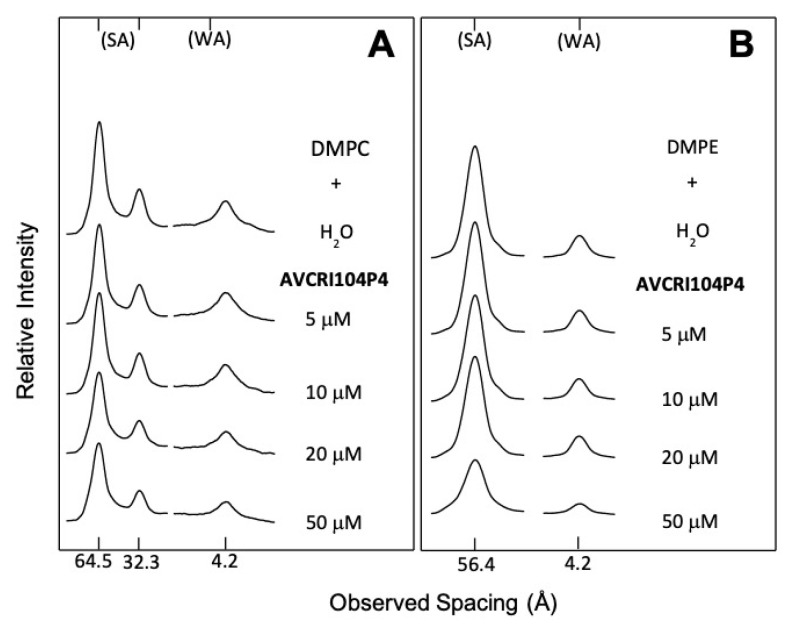
X-ray diffraction patterns of (**A**) dimyristoylphosphatidylcholine (DMPC) and (**B**) dimyristoylphosphatidylethanolamine (DMPE) in water and incubated with aqueous solutions of AVCRI104P4; (SA) small-angle and (WA) wide-angle reflections.

**Figure 4:**
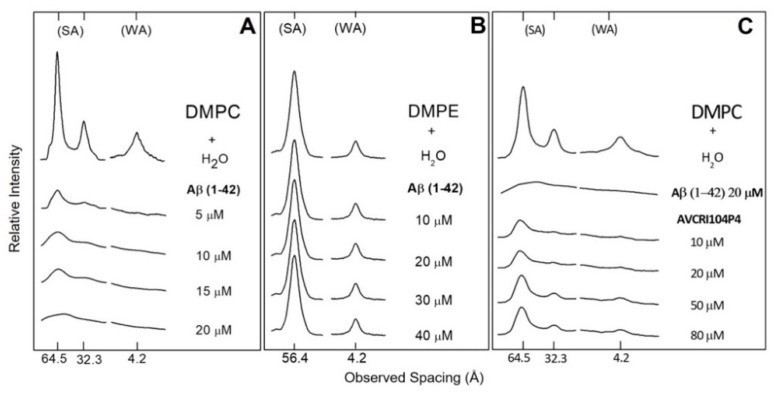
X-ray diffraction patterns of (**A**) dimyristoylphosphatidylcholine (DMPC) and (**B**) dimyristoylphosphatidylethanolamine (DMPE) in water and incubated with Aβ(1-42); (**C**) DMPC in water, and incubated with AVCRI104P4 and Aβ(1-42); (SA) small-angle and (WA) wide-angle reflections.

The diffraction patterns of DMPC in water and incubated with aqueous solutions of AVCRI104P4 in a concentration range of 5–50 µM are shown in Figure 3A. As expected, water altered the structure of DMPC, increasing its interlayer space from approximately 55 Å in its dry crystalline form to 64.5 Å when incubated in water, and its small-angle reflections (SA) that correspond to the polar head group separation were reduced to only the first two orders of the bilayer width. On the other hand, an isolated reflection at 4.2 Å located in the wide-angle region (WA) was also observed, corresponding to the mean distance between fully extended acyl chains organized with rotational disorder in hexagonal packing. These characteristics indicated the presence of a P_β′_ phase [44]. In the concentration range of 5–50 µM, AVCRI104P4 induced a gradual decrease in DMPC reflection intensities, which at the peptide maximum concentration reached 13.4% in the signal corresponding to the polar heads groups (SA), and 8.2% in the zone relative to hydrophobic chains (WA) (Table 1). On the other hand, the effect of AVCRI104P4 on DMPE multibilayers can be observed in Figure 3B. In this case, the peptide has a greater effect than on DMPC as at the 50 µM concentration the reflection intensities decreased by 41.3% and 43.6% for the polar head group and hydrophobic chain regions of the phospholipid, respectively (Table 1). Figure 4A shows the results of X-ray diffraction experiments of DMPC in water and of DMPC incubated with Aβ(1-42) in the 5–20 µM concentration range. According to these results, Aβ(1-42) was able to strongly decrease the normal diffraction intensities of DMPC both at the level of head polar groups and hydrophobic chains. This effect was observed even at a very low concentration (5 µM), where the diffraction intensity decreased by 81.3% in the area of head polar groups and 81.6% in that of the hydrophobic chains (Table 2). With increasing Aβ(1-42) concentrations, there was continuous weakening of reflection intensities, reaching an almost complete extinction at a 20 µM concentration (Table 2). The results of the X-ray diffraction studies of DMPE in water and DMPE incubated with Aβ(1-42) are presented in Figure 4B. In this system, the Aβ(1-42) peptide in the 10-40 µM concentration range had a slight effect on the arrangement of DMPE head polar groups and acyl chains. With the maximum studied concentration (40 µM), a very low decrease in the diffraction intensities was observed, being 3.0% for the head polar groups and 3.3% for the hydrophobic chain regions (Table 2). The study of the protective effect of the AVCRI104P4 hybrid in multilayers of DMPC against 20 µM Aβ(1-42) is presented in Figure 4C. As can be observed, 20 µM Aβ(1-42) induced a strong decrease in DMPC diffraction intensities of both SA and WA regions. However, when DMPC was first incubated with AVCRI104P4 in the 10–50 µM concentration range, the deleterious effect of the 20 µM Aβ(1-42) was considerably lowered and a recovery of the DMPC structure was progressively attained (Table 3).

### 2.3. Differential Scanning Calorimetry (DSC) of Multilamellar Vesicles (MLV) of DMPC and DMPE

**Figure 5:**
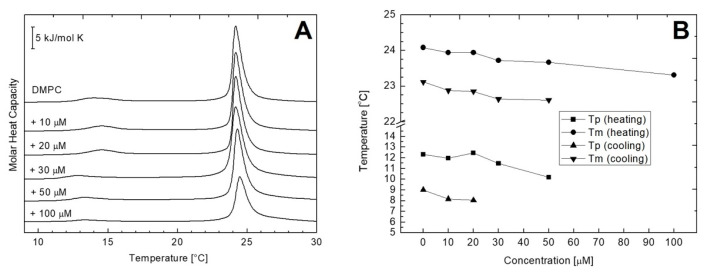
(**A**) Representative DSC curves obtained for multilamellar DMPC liposomes containing different AVCRI104P4 concentrations. Scans were recorded at a heating rate of 1 °C min^−1^; (**B**) a plot of phase transition temperature of DMPC multilamellar liposomes determined for cooling and heating scans as a function of AVCRI104P4 concentration.

**Figure 6:**
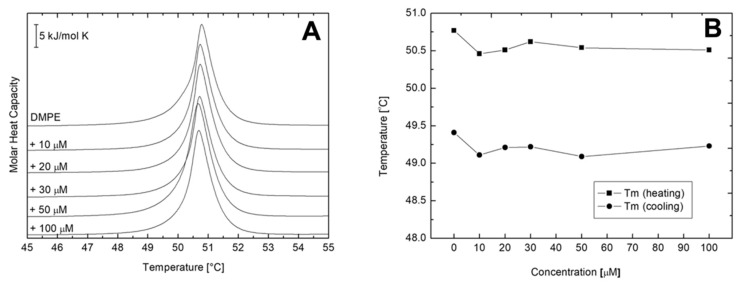
(**A**) Representative DSC curves obtained for multilamellar DMPE liposomes containing different AVCRI104P4 concentrations. Scans were recorded at a heating rate of 1 °C min^−1^; (**B**) a plot of phase transition temperature of DMPE multilamellar liposomes determined for cooling and heating scans as a function of AVCRI104P4 concentration.

Phospholipids are one of the most studied lipids by differential scanning calorimetry (DSC). Their behavior in response to controlled heating/cooling is considered as well-defined since while fully hydrated they undergo reproducible phase transitions at precisely determined temperatures. In general, a typical strong and sharp main transition occurs, which, in case of phosphatidylcholines, is additionally preceded by a weak signal corresponding to the pre-transition. As shown in Figure 5A, the fully hydrated DMPC bilayers in the absence of AVCRI104P4 showed in the temperature range of 0–30 °C the acute main transition at the temperature (T_m_) of 24.08 °C, with a ΔH of 19.93 kJ mol^−1^ arising from the conversion of the rippled gel phase (P_β′_) to the lamellar liquid crystal phase (L_α_) (Figure 5A, Table 4). At 12.31 °C, a pre-transition derived from the conversion of the lamellar gel phase (L_β’_) to the rippled gel phase (P_β′_) was observed, with ΔH of 1.98 kJ mol^−1^ (Table 4). These results are consistent with previous reports in the literature [45,46]. In Figure 5A, a set of representative heating thermographs that were obtained for MLV of pure DMPC and mixtures of DMPC and AVCRI104P4 in a concentration of 10 to 100 µM also is shown. After the addition of AVCRI104P4, the behavior of the thermotropic phase of DMPC changed slightly. Only at a high concentration of the compound (100 µM), a gradual decrease in the transition peak of the main phase and a small displacement of T_m_ at lower temperatures was observed (Figure 5B). In addition, AVCRI104P4 affected the pre-transition phase of DMPC, which disappeared completely at 100 µM. On the other hand, pure DMPE vesicles showed a single acute transition at 50.77 °C with an enthalpy change of 20.73 kJ mol^−1^ in the thermal range of 30–70 °C (Figure 6A, Table 5). This transition, described as the transformation from the gel phase (L_β_) to the lamellar liquid crystal phase (L_α_), was highly reproducible, strong, and sharp, with an almost symmetrical profile. Represented in Figure 6A, the heating profiles registered for the DMPE bilayers showed a considerably lower capacity of AVCRI104P4 to distort the phase transition of DMPE molecules. In Figure 6B, the values of the main transition and pre-transition temperatures are represented as a function of AVCRI104P4 concentration. As a general characteristic, in both heating and cooling processes in the presence of AVCRI104P4 no significant changes in the thermotropic behavior of DMPE vesicles were observed. The complete data sets of thermodynamic parameters, including temperature, entropy, and enthalpy values for these series of experiments, are shown in Table 4 and Table 5 for DMPC and DMPE, respectively.

### 2.4. Scanning Electron Microscopy (SEM) Analysis on Human Erythrocytes

**Figure 7:**
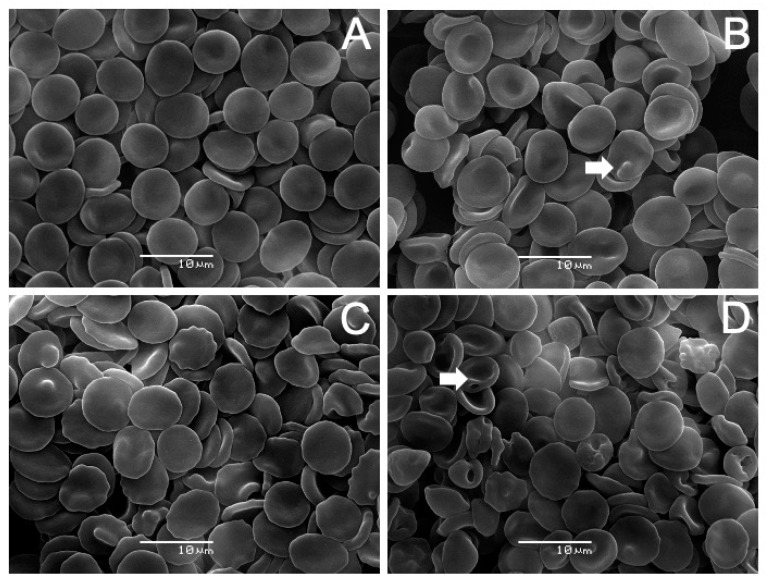
AVCRI104P4 effects on the morphology of human erythrocytes. Images obtained by scanning electron microscopy (SEM) of (**A**) Control, (**B**) 10 μM, (**C**) 30 μM, and (**D**) 50 μM AVCRI104P4. Arrows in Figure 7B,D highlight an echinocyte and a stomatocyte, respectively.

**Figure 8:**
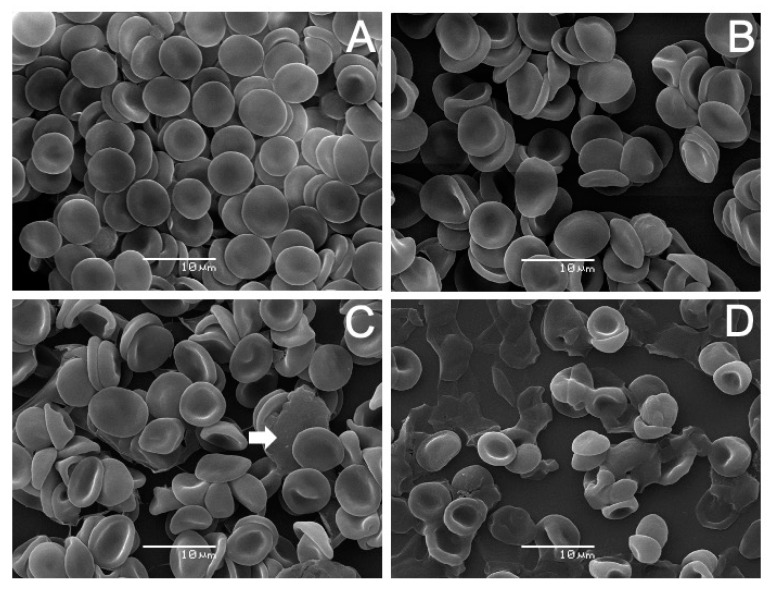
Aβ(1-42) effect on the morphology of human erythrocytes. Images obtained by scanning electron microscopy (SEM) of (**A**) Control, (**B**) 5μM, (**C**) 10 μM, and (**D**) 20 μM Aβ(1-42). Arrow in Figure 8C highlights an erythrocyte membrane fragment.

**Figure 9:**
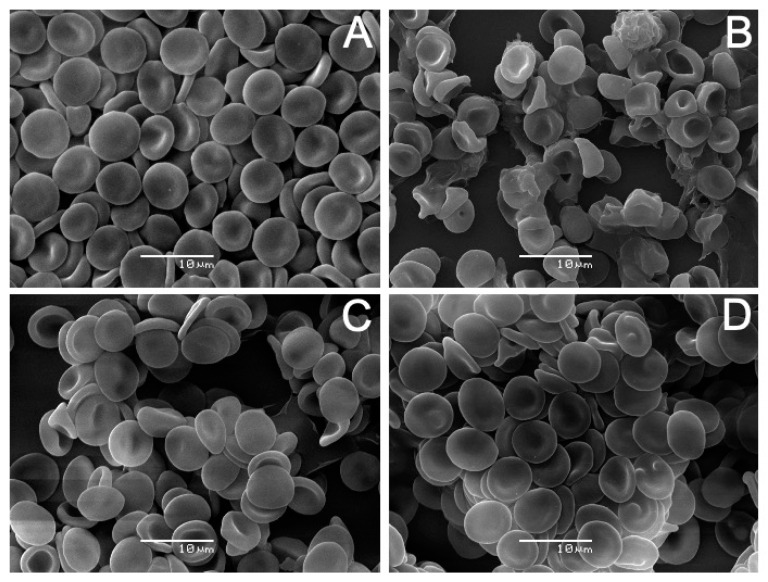
AVCRI104P4 protective effect on human erythrocytes. SEM images of (**A**) untreated erythrocytes; incubated with (**B**) 20 μM Aβ(1-42); (**C**) 10 μM AVCRI104P4 and 20 μM Aβ(1-42); (**D**) 20 μM AVCRI104P4 and 20 μM Aβ(1-42).

The results of incubating erythrocytes with AVCRI104P4 are shown in Figure 7. The analysis revealed that human erythrocytes treated with AVCRI104P4 in the 10–50 µM concentration range experienced noticeable changes on their surface modifying their normal discocyte shape (Figure 7A, Control). In the presence of 10 µM AVCRI104P4 (Figure 7B), a low percentage (7.9%) of echinocytes was observed. These cell forms are characterized by the presence of spicules on the cell surface (arrow). At 30 µM (Figure 7C), the hybrid produced echinocytosis in more than 50% of the cells (78.4%). With 50 µM AVCRI104P4 (Figure 7D), a large number (78.7%) of stomatocyte deformed cells are observed (arrow), characterized by presenting an invagination on their surface showing a cup shape. Figure 8 shows the effects of Aβ(1-42) on human erythrocytes. With low concentrations (5 µM, Figure 8B), this molecule induced the presence of echinocytes and stomatocytes, the latter in the highest number (71.8%). Figure 8C shows that almost all the the cells (>80%) have a stomatocytic morphology; in addition, there are signs of cell lysis (arrow). A pronounced change in the erythrocyte morphology accompanied by cell lysis was induced by a higher peptide concentration (20 µM, Figure 8D); the remains of destroyed erythrocyte membranes can be observed. The results of the study of the protective capacity of the AVCRI104P4 hybrid against the effect of 20 µM Aβ(1-42) on erythrocytes are shown in Figure 9. As can be seen in Figure 9B, 20 µM Aβ(1-42) induced an alteration of the discocytic form of red blood cells, generating stomatocytosis and lysis. When erythrocytes were previously incubated with AVCRI104P4 and the Aβ(1-42) peptide was added, the peptide-induced alteration was reversed. Cells incubated with 10 µM AVCRI104P4 still show a large number (55.6%) of stomatocytes (Figure 9C), but some normal cells (discocytes) are also observed (43.2%), whose number increased with 20 µM AVCRI104P4, reaching 70.4% of the total number of cells observed (Figure 9D). These results demonstrate the protective effect of AVCRI104P4 against the deleterious effect of 20 µM Aβ(1-42). In the Supplementary Material section Figures S1–S3 are shown which detail the population distribution of cells observed by SEM. In the same section the percentages of cells observed at each concentration are shown (Table S1).

## 3. Discussion

In order to elucidate the molecular mechanisms of the interaction of the multitarget hybrid compound AVCRI104P4 and cell membranes and to determine the possible protective effect of the hybrid against the toxic effect of Aβ(1-42) peptide, human erythrocytes and molecular models of its membrane were used. The models consisted in dimyristoylphosphatidylcholine (DMPC) and dimethiphosphatidylamine (DMPE), which correspond to phospholipids representing classes located in the outer and inner monolayers of the erythrocyte membrane, respectively. DMPC and DMPE differ only in their amino terminal groups, which are ^+^N(CH_3_)_3_ in DMPC and ^+^NH_3_ in DMPE.

X-ray diffraction results showed that AVCRI104P4 exhibited a moderate effect on DMPC structure. At the maximum concentration of this hybrid (50 µM), a reduction in the reflection intensity of 13.4% was observed in the area of the head polar groups and 8.2% in the area of the hydrophobic chains (WA). However, at the same concentration its effect was more pronounced in DMPE, decreasing the reflection intensity by 41.3% and 43.6% in the SA and WA zones, respectively. DMPE molecules are packaged in a more compact form than DMPC molecules due to the smaller size of the polar head group and the resulting higher effective charge. This leads to a more stable formation with strong electrostatic and hydrogen bonding interactions that are not easily affected by water [47]. On the other hand, the interactions between neighboring DMPC layers are weaker due to their bulky head groups. This allows water to fill the polar spaces between layers resulting in an increase in their separation [48]. Notwithstanding that, AVCRI104P4 had the ability to preferentially alter the DMPE structure in both areas of the phospholipid. This may be due to electrostatic interactions between the charges of DMPE and those of AVCRI104P4. At pH 7.4, the predominant form of DMPE is zwitterionic, which presents a negative charge in one phosphate oxygen, and a positive charge in its primary amine group [49]. Therefore, either of the two basic nitrogen atoms that possess AVCRI104P4 (which are positively charged at pH 7.4; Figure 1) would interact with the DMPE phosphate group orienting the zone of the methoxy groups (R-OMe) towards the interlayer region generating the signals observed by X-ray diffraction. This interaction would also be theoretically possible in the case of DMPC. However, this phospholipid contains larger methyl groups in the area of the polar heads, which would make the interaction of AVCRI104P4 with the negative charges of the phosphate groups sterically difficult.

DSC is one of the most successful techniques to study the thermotropic behavior of different compounds and biochemical interactions. The data obtained from the DSC experiments showed that AVCRI104P4 induced a pronounced change in the DMPC pre-transition zone (T_p_) towards smaller values (ΔT = 2.12 °C on heating) (Figure 5A and Table 4). This result indicates that the hybrid was capable of disturbing the transformation of DMPC typical lamellar gel (L_β’_) to the rippled gel phase (P_β′_). This phenomenon was accompanied by a decrease in the cooperation in the transition of the main phase, reflected in a decrease in its ΔH. This effect is related to the number of acyl chains involved in the conversion from the wavy gel phase (P_β′_) to the liquid crystalline phase (L_α_) [50], indicative that AVCRI104P4 altered the acyl chains’ order. This phenomenon was also seen in the experiments with DMPE, where the decrease in the cooperativity in the main phase transition was considerable, although it was not accompanied by a temperature shift. These results support the findings obtained by X-ray diffraction and confirm that AVCRI104P4 has a moderate effect on the ordering of both DMPC and DMPE molecules, mostly in the hydrophobic tails area. This could also be explained taking into account the hydrophobic nature of AVCRI104P4, which at pH = 7.4 has a partition coefficient (logP) of 7.231 and a distribution coefficient (logD) of 4.60 [51], which would indicate the affinity of the molecule for the lipid acyl chains. It should be mentioned that this is the first time that the interaction of AVCRI104P4 with lipid bilayers or molecular models of cell membranes has been reported.

The results of the SEM experiments showed that AVCRI104P4 induced morphological alterations in human erythrocytes from their normal discoid form to echinocytes and stomatocytes. Considering the bilayer couple hypothesis [52], the shape changes induced in human erythrocytes by extraneous molecules are due to a differential expansion of the two monolayers of the red blood cell membrane. When exogenous molecules insert in the outer moiety, echinocytes are produced, whereas stomatocytes are formed when the molecules locate into the inner monolayer of the membrane. The finding that AVCRI104P4 induced the formation of echinocytes and stomatocytes indicates that the hybrid was located preferentially in the outer monolayer of the membrane at low concentrations (10–30 μM) and in the inner monolayer of the cells at higher concentrations (50 μM), generating both types of morphological changes. It is important to note that in our in vitro studies on human erythrocytes, the used concentrations are considerably lower than those reported in the literature in experiments performed in bacteria [34,53]. The results obtained from X-ray diffraction and DSC studies support this conclusion, as they showed that AVCRI104P4 interacted with DMPC and DMPE, representative of phospholipids present in the outer and inner monolayers of the red cell membrane, respectively. There are no previous reports concerning the in vitro interaction of AVCRI104P4 with human erythrocytes, and information concerning its interaction with other cell types is minimal. However, AVCRI104P4 has been reported to have a high affinity for the enzyme AChE [33,34,35], which is abundant in the plasma membrane of human erythrocytes. This enzyme is linked to the erythrocyte membrane by an anchor of glycosylphosphatidylinositol (GPI) [54]. The AVCRI104P4 molecule consists mainly of two portions of potent AChE inhibitors: one related to donepezil, and the other to huprine Y (Figure 1). The carbonyl group of donepezil could form a strong hydrogen and water bond with the Ser286 residue of AChE [22], and the oxygen of the two methoxy groups may interact with the Trp279 residue at π-π [55]. Donepezil has also been reported to induce stomatocyte formation in human erythrocytes in the 20–40 μM concentration range [56]. All this evidence may provide insight into the molecular mechanism by which AVCRI104P4 induced the shape changes in erythrocytes. The possible binding sites of AVCRI104P4 with the human AChE enzyme have been modeled by molecular dynamics and have been described in detail [35].

In order to study the protective effect of AVCRI104P4, molecular models of cell membranes built of DMPC and DMPE bilayers and human erythrocytes were used, which were exposed to different concentrations of Aβ(1-42). A widely accepted hypothesis is that interactions between Aβ aggregates and neuronal membranes play an important role in toxicity [16,57,58,59,60]. In fact, it has been proposed that Aβ–membrane interactions induce alterations in membrane fluidity [61,62], production of free radicals, lipid peroxidation [63], formation of ion channels [64,65], changes in lipid metabolism, and an increase in phospholipase activity [66]. We therefore considered that it was of interest to understand the molecular mechanism of the interaction of Aβ(1-42) with cell membranes, and to examine the protective effect of AVCRI104P4. A common explanation is that Aβ(1-42) has an extracellular location, and that both Aβ(1-40) and Aβ(1-42) molecules interact strongly with negatively charged lipids [67,68]. Different reports state that Aβ(1-42) would be located in the hydrophobic nucleus of the membrane [69,70]. Previous X-ray diffraction studies reported that Aβ(1-42) produces structural alterations in multibilayers of DMPC and a moderate effect on DMPE [71,72]. These results are consistent with our current experimental findings. In fact, our X-ray diffraction studies showed that as the Aβ(1-42) concentration increases, a greater degree of disorder was observed in DMPC bilayers. The interactions of Aβ(1-42) with DMPE were comparatively milder as there was no marked effect on the structure of the phospholipid.

The protective effect of AVCRI104P4 against the toxic effect of Aβ(1-42) is presented in Figure 4C. As shown in the X-ray diffractograms, increasing concentrations of AVCRI104P4 neutralize the disruptive effect of Aβ(1-42) on DMPC bilayers. On the other hand, the results obtained by SEM for human erythrocytes incubated with Aβ(1-42) (Figure 9B) show that the peptide induced the formation of stomatocytes accompanied by cell lysis. This result can be attributed to the high toxicity of Aβ(1-42) in addition to its capacity to form pores on the cell surface [59,73]. However, pre-incubation of erythrocytes with AVCRI104P4 in increasing concentrations prevents the morphological alterations of red blood cells and cell lysis.

**Figure 10:**
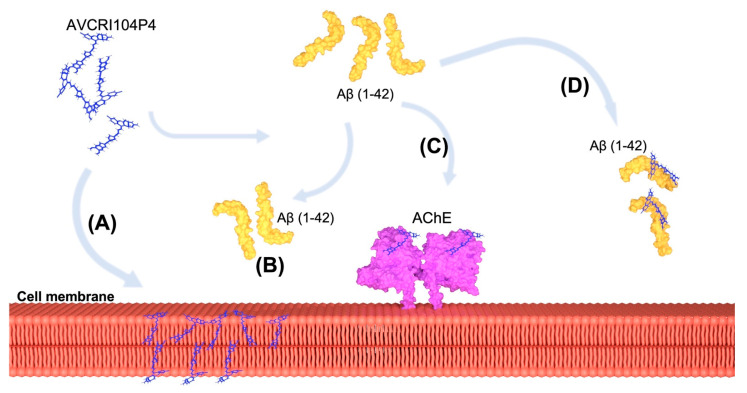
Representative schematic of the possible routes of action of AVCRI104P4. (**A**) AVCRI104P4 is able to interact with both monolayers of the cell membrane, so it can probably have a protective role at this position against the toxic effect of peptide Aβ (1-42) (represented by pathway (**B**). Pathway (**C**) illustrates the binding capacity of AVCRI104P4 to the enzyme AChE, which has a demonstrated Aβ (1-42)-aggregatory capacity. Finally, pathway (**D**) outlines the binding of AVCRI104P4 to Aβ (1-42), which would prevent its binding to the lipid membrane.

Taking into account our experimental results, it is possible to conclude that there may be different mechanisms through which AVCRI104P4 can protect the plasma membrane from the toxic effects of Aβ(1-42) (Figure 10). The fact that the hybrid has the ability to bind to both DMPC and DMPE types of phospholipids would indicate one mechanism to protect the membrane (Figure 10A). On the other hand, the dual effect of AVCRI104P4 of binding both AChE and avoiding AChE-induced aggregation of Aβ(1-42) might also be a mechanism of cell membrane protection (Figure 10C). Another possible mechanism that has not yet been explored is the direct interaction between free AVCRI104P4 molecules and Aβ(1-42) (Figure 10D). The knowledge of this mechanism would lead to a more precise identification of the Aβ(1-42) residues that interact with the membrane lipids. In summary, our results showed that ACRI104P4 is capable of interacting with both types of phospholipids representative of cell membranes (DMPC and DMPE) and also with human erythrocytes, neutralizing the deleterious effects of the peptide Aβ(1-42) on these cells.

## 4. Materials and Methods

### 4.1. Chemicals

AVCRI104P4 [(-)-(7S,11S)-3-chloro-12-[(3-{4-[(5,6-dimethoxyindan-2-yl)methyl]piperidin-1-yl}propyl)amino]-6,7,10,11-tetrahydro-9-methyl-7,11-methanocycloocta[b]quinoline, C_37_H_46_ClN_3_O_2_, *M*_W_ 600.2] was synthesized following the experimental procedure detailed in Viayna et al. [35]. Aβ(1-42) (C_203_H_311_N_55_O_60_S, *M*_W_ 4514.1, purity >95%) was from GenicBio (Shanghai, China); dimyristoylphosphatidylcholine (DMPC, *M*_W_ 677.9) and dimyristoylphosphatidylethanolamine (DMPE; *M*_W_ 635.9) were from Aldrich (Milwaukee, WI, USA).

### 4.2. Preparation of Oligomeric Aggregates of Aβ(1-42)

Aβ(1-42) oligomers were prepared following the protocol of Peters et al. [74]. The lyophilized powder form of Aβ(1-42) was dissolved in hexafluoroisopropanol (HFIP), then aliquoted, evaporated at room temperature, and stored at –20 °C. For both X-ray diffraction experiments and scanning electron microscopy (SEM) observations, oligomeric aggregates of Aβ(1-42) were used. For the preparation of oligomers, ultrapure water was added to the aliquots in an Eppendorf tube to a final concentration of 80 µM. After 20 min incubation at room temperature, the samples were stirred at 400 rpm using a Teflon-coated micro-stir bar for 24–48 h at room temperature (∼20 °C) and subsequently stored at 4 °C until required. In order to observe the capacity of the oligomeric aggregates to form fibers, they were incubated at 37 °C for 24 h (Thermo Haake C10, Dreieich, Germany).

### 4.3. Transmission Electron Microscopy of Aβ(1-42) Oligomeric Aggregates and Fibers

In order to observe the ultrastructure of the oligomeric aggregates and fibers used in the subsequent experiments, an aliquot of 10 µL of 80 µM Aβ(1-42) was placed on a carbon-coated Formvar grids and then fixed with a 2% glutaraldehyde solution for 5 min. The Aβ(1-42) aggregates were stained with 5 µL of 0.2% (*w*/*v*) phosphotungstic acid (PTA) and then dried at room temperature. The samples were examined with a JEOL 1200 EX II electron microscope [74].

### 4.4. X-ray Diffraction of DMPC and DMPE Multibilayers

The sample preparation was the same for both DMPC and DMPE phospholipids. A blank, containing about 2 mg (Cahn C-33 Microbalance, Beverly, Orion, USA) of the respective phospholipid, was prepared in Eppendorf tubes to which 200 µL of bi-distilled water was added. Subsequently, about 2 mg of each phospholipid was placed in Eppendorf tubes, and 200 µL of aqueous solutions of AVCRI104P4 and Aβ(1-42) were added in different concentrations to each of DMPC and DMPE. The blank and the tubes with the phospholipids plus the compounds were then incubated for 30 min in a water bath with an immersion thermostat (Thermo Haake C10, Dreieich, Germany) at 37 °C for DMPC and 60 °C for DMPE. In order to study the protective effect of AVCRI104P4, this compound was incubated with DMPC for 30 min at 37 °C and then for 30 min with 20 μM Aβ(1-42). Once the incubation was finished, each suspension was placed in a special glass capillary (Glas-Technick&Konstruction, Berlin, Germany) and centrifuged at 2500 rpm for 15 min (Centrifuga Selecta, Mod. Mixtasel, Spain) to finally take the samples to the X-ray generator (Bruker Kristalloflex 760, Berlin, Germany) and diffracted using a CuKα (*λ* = 1.52 Å) radiation with Ni filter at a controlled room temperature of 18 ± 2 °C. The intensities and interplanar spacings were obtained through ASA software attached to the detector (Hecus M. Braun PSD 50M, Garching, Germany). The Origin 8.0 program (OriginLab Corporation, Northampton, MA, USA) was used for the analysis and treatment of the data (areas under the curve). Each experiment was carried out in triplicate.

### 4.5. Differential Scanning Calorimetry (DSC) of Multilamellar Vesicles (MLV) of DMPC and DMPE

Appropriate amounts of DMPC or DMPE dissolved in pure chloroform (analytical quality) were prepared in a glass test tube to obtain a final phospholipid concentration of 1 mM. A gentle flow of nitrogen was used to remove the solvent and form a thin film on the walls of the glass tubes. The films were hydrated with distilled water (and aqueous solutions of the compound under study), and multilamellar vesicles (MLV) were formed by vortexing the samples for 1 min at a temperature higher than the phase transition temperature of the pure phospholipid chain (approximately 30 °C and 60 °C for DMPC and DMPE, respectively). The DSC experiments were performed using the NANO DSC Series III platinum capillary cell system (TA Instruments, New Castle, DE, USA) with an active volume of 300 μL. The samples were degassed to prevent bubble formation by pulling a vacuum of 30.4–50.7 kPa in the solution for 15 min. Then, 300 μL of sample solution was placed in the sample cell and an equal volume of distilled water was used as a reference. The cells were sealed and thermally balanced for 10 min at the starting temperature. Calorimetric analyses were carried out on samples with a pressure of 0.3 MPa. The heating/cooling rates were 1 °C min^−1^, and the scans were recorded within a range of 5–40 °C (DMPC) and 30–70 °C (DMPE). The thermograms were corrected by subtracting blank water scans and normalizing to the corresponding lipid concentration. The thermodynamic parameters were obtained using the TA Instruments software package. The DSC measurements were performed in triplicate.

### 4.6. Scanning Electron Microscopy (SEM) of Human Erythrocytes

In vitro studies of the effects of the compound under study on human erythrocytes were carried out by observing changes in their morphology. For this purpose, blood was obtained from a healthy adult donor without pharmacological treatment. Approximately one drop of blood was received in an Eppendorf tube with 1000 µL PBS (Phosphate Buffer Saline) 1 × pH 7.4 with 1 mg/mL BSA (Bovine Serum Albumin). The sample was centrifuged at 1000 rpm for 10 min (Centrifuga Selecta, Mod. Mixtasel, Spain); the supernatant was extracted and replaced with an equal amount of PBS/BSA solution. This procedure was repeated three times to ensure complete removal of the plasma. The red blood cell sample was distributed in Eppendorf tubes, including a control. The supernatant was centrifuged and replaced by 250 µL of a solution of the compound under study in different concentrations prepared in PBS/BSA. The samples were then incubated at 37 °C for 1 h in a water bath with an immersion thermostat (Thermo Haake C10, Dreieich, Germany), and then centrifuged at 1000 rpm for 10 min. To evaluate the protective effect of AVCRI104P4, erythrocytes were pre-incubated with this compound (1–20 µM) for 30 min and then with Aβ(1-42) 20 µM for 30 min. After centrifugation, the supernatant was removed and replaced with 250 µL of 2.5% glutaraldehyde and left to stand for 24 h at 4 °C. After this, the samples were centrifuged at 1000 rpm for 10 min, the supernatant was removed, and 500 µL of nanopure water was added. In order to eliminate the glutaraldehyde from the erythrocytes, the procedure was performed three more times. Subsequently, approximately 10 µL of each sample was deposited on a thin glass plate on an aluminum support, it was left to dry in an oven at 37 °C and then metalized with a gold bath at 13.3 Pa for 3 min (Edwards S150, Sussex, England). Then, the samples were observed in the scanning electron microscope (JEOL, Mod. JSM 6380 LB, Tokyo, Japan). Percentage determinations were obtained by counting approximately 300 cells from three sample observations of each concentration at 2500× magnification.

## Figures and Tables

**Table 1:** Decrease in the area under the curve (%) in the (SA) small-angle and (WA) wide-angle reflections of DMPC and DMPE by the effect of AVCRIP104P4. Percentages obtained from the analysis of microdensitograms from X-ray diffraction patterns of DMPC and DMPE in water and incubated with AVCRI104P4 solutions.

AVCRI104P4	Area Under Curve Decrease (%)			
Concentration [µM]	DMPC		DMPE	
	SA	WA	SA	WA
5	9.5 ± 0.4	4.6 ± 0.2	4.2 ± 0.1	15.7 ± 0.7
10	10.0 ± 0.5	4.7 ± 0.8	11.7 ± 0.3	21.4 ± 0.3
20	11.1 ± 0.7	5.2 ± 0.9	26.7 ± 0.8	34.5 ± 0.7
50	13.4 ± 0.7	8.2 ± 0.3	41.3 ± 1.1	43.6 ± 0.9

**Table 2:** Decrease in the area under the curve (%) in the (SA) small-angle and (WA) wide-angle reflections of DMPC and DMPE by the effect of Aβ(1-42). Percentages obtained from the analysis of microdensitograms from the X-ray diffraction patterns of DMPC and DMPE in water and incubated with Aβ(1-42) solutions.

Aβ (1-42)	Area Under Curve Decrease (%)			
Concentration [µM]	DMPC		DMPE	
	SA	WA	SA	WA
5	83.1 ± 0.4	83.6 ± 0.9	2.1 ± 0.2	1.7 ± 0.1
10	92.1 ± 1.5	99.9 ± 0.1	2.9 ± 0.1	3.1 ± 0.1
20	94.5 ± 1.6	99.9 ± 0.6	2.8 ± 0.4	3.2 ± 0.4
30	99.9 ± 0.9	99.9 ± 0.3	3.0 ± 0.2	3.3 ± 0.2

**Table 3:** Decrease in the area under the curve (%) in the (SA) small-angle and (WA) wide-angle reflections of DMPC and DMPE by the effect of 20 µM Aβ(1-42) and AVCRI104P4 solutions. Percentages obtained from the analysis of microdensitograms from X-ray diffraction patterns of DMPC and DMPE in water and incubated with 20 µM Aβ(1-42) and AVCRI104P4 solutions.

	Area under Curve Decrease (%)	
Concentration [µM]	DMPC	
	SA	WA
Aβ(1-42) 20 µM	99.3 ± 0.3	99.1 ± 0.4
+AVCRI104P4		
10 µM	81.4 ± 0.9	95.2 ± 0.1
20 µM	73.6 ± 0.3	94.6 ± 0.5
50 µM	64.2 ± 0.7	92.1 ± 0.1
80 µM	61.7 ± 0.5	89.5 ± 0.7

**Table 4:** Thermodynamic parameters of pre-transition and main phase transition of pure, fully hydrated, multilamellar liposomes and mixtures of DMPC/AVCRI104P4 obtained from heating and cooling; scans collected at a rate of 1 °C min^−1^ for both processes. The accuracy of the main phase transition temperature and enthalpy was ±0.01 °C and ±0.8 kJ/mol, respectively.

**AVCRI104P4 Concentration [μM]**	**Pre-Transition** **(Heating)**			**Main Transition** **(Heating)**		
**DMPC +** **AVCRI104P4**	**ΔH** **[kJ/mol]**	**ΔS** **[J/mol K]**	**Tp** **[°C]**	**ΔH** **[kJ/mol]**	**ΔS** **[J/mol K]**	**Tm** **[°C]**
0	1.98	0.67	12.31	13.01	6.01	24.08
10	0.78	0.51	11.96	15.05	5.07	23.94
20	0.35	0.20	12.45	14.35	4.83	23.94
30	0.32	0.11	11.48	11.96	4.03	23.72
50	0.23	0.12	10.19	12.78	4.31	23.67
100	-	-	-	9.91	3.34	23.31
**AVCRI104P4 Concentration [μM]**	**Pre-Transition** **(Cooling)**			**Main Transition** **(Cooling)**		
**DMPC +** **AVCRI104P4**	**ΔH** **[kJ/mol]**	**ΔS** **[J/mol K]**	**Tp** **[°C]**	**ΔH** **[kJ/mol]**	**ΔS** **[J/mol K]**	**Tm** **[°C]**
0	1.03	0.35	9.01	20.32	6.61	23.12
10	0.09	0.03	8.17	14.92	5.04	22.88
20	0.04	0.10	8.05	13.21	4.46	22.85
30	-	-	-	12.14	4.11	22.64
50	-	-	-	11.73	4.64	22.61
100	-	-	-	10.10	3.79	19.88

**Table 5:** Thermodynamic parameters of pre-transition and main phase transition of pure, fully hydrated, multilamellar liposomes and mixtures of DMPE/AVCRI104P4 obtained from heating and cooling scans collected at a rate of 1 °C min^−1^ for both processes. The accuracy of the main phase transition temperature and enthalpy was ±0.01 °C and ±0.8 kJ/mol, respectively.

AVCRI104P4 Concentration [μM]	Main Transition (Heating)			Main Transition (Cooling)		
DMPE + AVCRI104P4	ΔH [kJ/mol]	ΔS [J/mol K]	Tm [°C]	ΔH [kJ/mol]	ΔS [J/mol K]	Tm [°C]
0	20.73	6.01	50.77	19.17	6.06	49.41
10	14.97	4.64	50.46	17.18	4.13	49.11
20	13.16	4.07	50.51	17.84	5.81	49.21
30	10.51	3.25	50.62	18.43	4.52	49.22
50	12.46	3.86	50.54	18.53	6.23	49.09
100	6.49	2.01	50.51	19.17	5.18	49.23

## Supplementary Materials

The following are available online at https://www.mdpi.com/article/10.3390/ijms22179563/s1.
