# Supplementary material for: Protective Role of a Donepezil-Huprine Hybrid against the β-Amyloid (1-42) Effect on Human Erythrocytes
Source: Int J Mol Sci. 2021 Sep 3;22(17):9563. doi: 10.3390/ijms22179563 (PMC8431064; doi:10.3390/ijms22179563)
Supplement: Supplementary file 1 [file ijms-22-09563-s001.zip › ijms-1349294-supplementary.pdf]

International Journal of
Molecular Sciences

MDPI

Article

Protective role of a donepezil-huprine hybrid against the β-amyloid (1-42) effect on human erythrocytes

Pablo Zambrano [1,*,†], Mario Suwalsky [2], Malgorzata Jemiola-Rzeminska [3,4], María José Gallardo-Nelson [5], Kazimierz Strzalka [3,4] and Diego Muñoz-Torrero [6,7].

[1] Facultad de Ciencias Químicas, Universidad de Concepción, 4030000, Concepción, Chile
[2] Facultad de Medicina, Universidad Católica de la Santísima Concepción, 4030000, Concepción, Chile; msuwalsky@ucsc.cl
[3] Malopolska Centre of Biotechnology, Jagiellonian University, 30-387, Kraków, Poland; malgorzata.jemiola-rzeminska@uj.edu.pl(M.J.-R.), kazimierz.strzalka@uj.edu.pl(K.S.)
[4] Faculty of Biochemistry, Biophysics and Biotechnology, Jagiellonian University, 30-387, Kraków, Poland
[5] School of Medicine, University of Atacama, 1530000, Copiapó, Chile; mariajose.gallardo@uda.cl
[6] Laboratory of Medicinal Chemistry (CSIC Associated Unit), Faculty of Pharmacy and Food, Sciences, University of Barcelona (UB), E-08028 Barcelona, Spain; dmunoztorrero@ub.edu
[7] Institute of Biomedicine (IBUB), University of Barcelona (UB), E-08028 Barcelona, Spain
* Correspondence: E-mail address: pzambranol@udec.cl, Tel: +49 89 8578-2374
† Current address: Max Planck Institute of Biochemistry, Am Klopferspitz 18, 82152 Martinsried, Germany

Supplementary Materials

Table S1. Percentages obtained from scanning electron microscopy observations of the population distribution of human erythrocytes incubated with different concentrations of AVCRI104P4, Aβ(1-42) and protective effect of AVCRI104P4 against Aβ(1-42) 20 μM.

	**Echinocytes**	**Stomatocytes**	**Discocytes**
Control	0.0	1.1	98.9
AVCRI104P4 10 μM	5.9	11.8	82.4
AVCRI104P4 30 μM	78.4	20.6	0.0
AVCRI104P4 50 μM	21.3	78.7	0.0
	Echinocytes	Stomatocytes	Discocytes
Control	0.0	0.9	96.4
Aβ(1-42) 5 μM	10.0	71.8	0.0
Aβ(1-42) 10 μM	2.7	88.2	0.0
Aβ(1-42) 20 μM	0.0	11.8	0.0
	Echinocytes	Stomatocytes	Normocytes
Control	0.9	2.8	92.5
Aβ(1-42) 20 μM	0.0	33.3	4.4
AVCRI104P4 10 μM + Aβ(1-42) 20 μM	1.2	55.6	43.2
AVCRI104P4 20 μM + Aβ(1-42) 20 μM	4.6	25.0	70.4

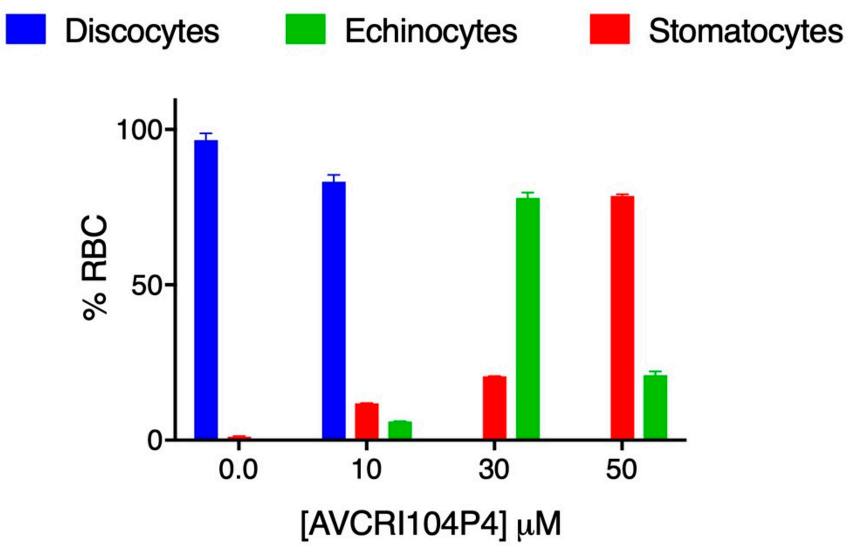

Figure S1. Scanning electron microscopy observations of population distribution of human erythrocytes incubated with different concentrations of AVCRI104P4 (n = 3). Values are the mean ± SD.

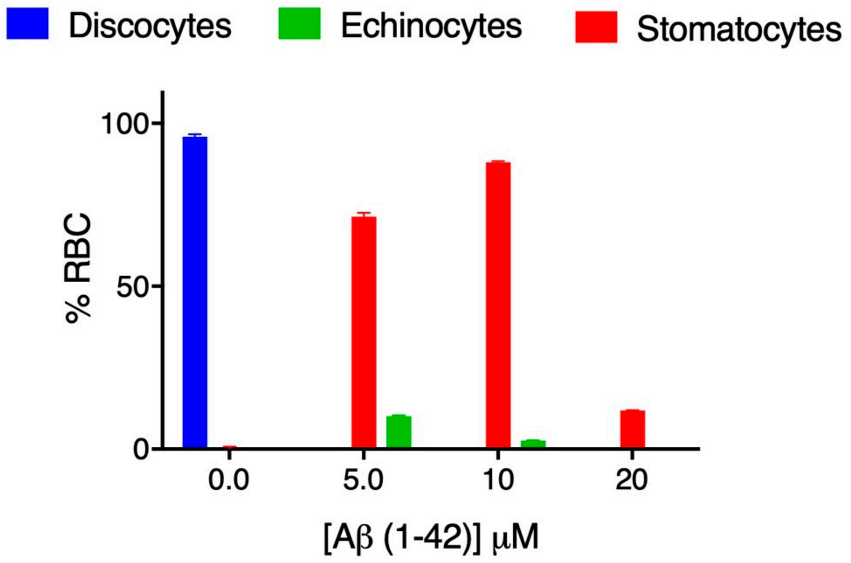

Figure S2. Scanning electron microscopy observations of population distribution of human erythrocytes incubated with different concentrations of Aβ(1-42) (n = 3). Values are the mean ± SD.

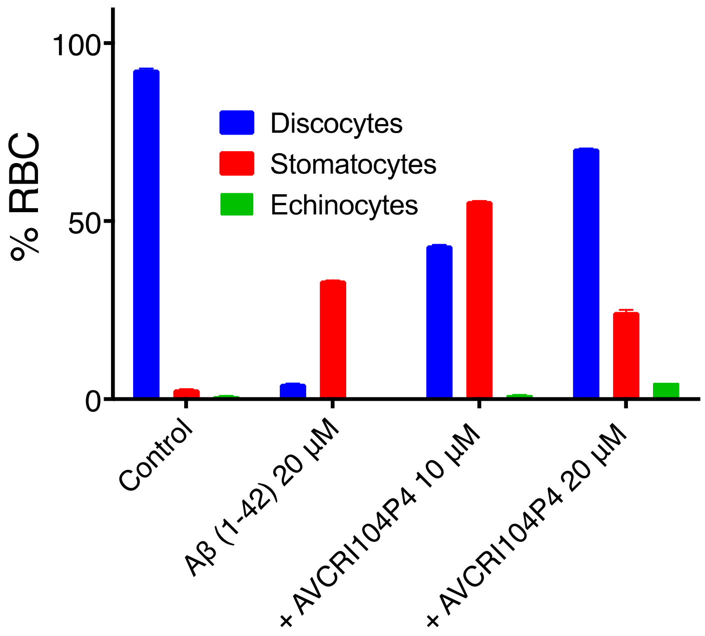

Figure S3. Scanning electron microscopy observations of population distribution of human erythrocytes incubated with different concentrations of AVCRI104P4 and exposed to Aβ(1-42) 20 μM (n = 3). Values are the mean ± SD.
